# Don’t let sleeping dogs lie: unravelling the identity and taxonomy of *Babesia canis*, *Babesia rossi* and *Babesia vogeli*

**DOI:** 10.1186/s13071-020-04062-w

**Published:** 2020-04-21

**Authors:** Barend L. Penzhorn

**Affiliations:** 1 Vectors and Vector-borne Diseases Research Programme, Department of Veterinary Tropical Diseases, Faculty of Veterinary Science, University of Pretoria, Private Bag X04, Onderstepoort, 0110 South Africa; 2 Research Associate, National Zoological Gardens, South African National Biodiversity Institute, P.O. Box 724, Pretoria, 0001 South Africa

**Keywords:** *Babesia canis*, *Babesia rossi*, *Babesia vogeli*, Canine babesiosis, *Dermacentor reticulatus*, *Haemaphysalis elliptica*, History, *Rhipicephalus sanguineus*

## Abstract

For most of the 20th century the causative agent of canine babesiosis, wherever it occurred in the world, was commonly referred to as *Babesia canis*. Early research, from the 1890s to the 1930s, had shown that there were three distinctly different vector-specific parasite entities occurring in specific geographical regions, that host response to infection ranged from subclinical to acute, and that immunity to one stock of the parasite did not necessarily protect against infection with other stocks. This substantial body of knowledge was overlooked or ignored for 50 years. In this review the first records and descriptions of the disease in four geographical regions were traced: sub-Saharan Africa, Europe, North Africa and Asia. Research leading to identification of the specific tick vector species involved is documented. Evidence is given of the growing realisation that there were substantial biological differences between stocks originating from different geographical regions. Etymological provenance for *Babesia vogeli* is proposed.
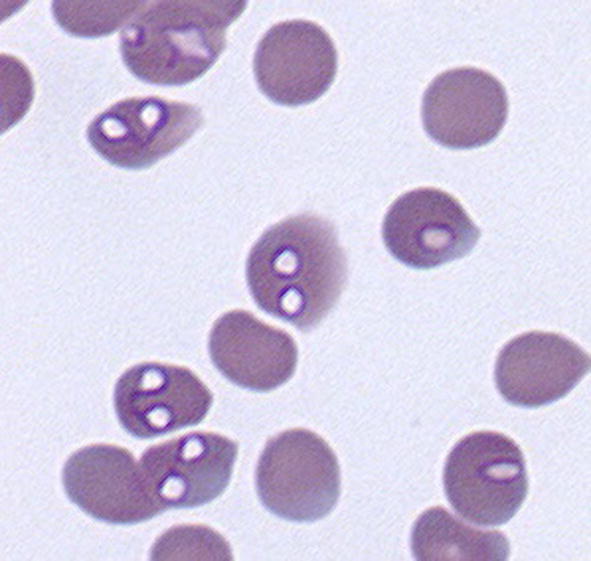

## Background

Babesiosis, a tick-transmitted disease affecting dogs in many parts of the world, is caused by various *Babesia* spp. *Babesia canis* (Piana & Galli-Valerio, 1895) (*sensu stricto*) was described and named in 1895 in northern Italy [1]. During the next three decades, work by numerous researchers indicated clearly that there were three distinct, vector-specific parasite entities lumped under the name *B. canis*. Clinical manifestation of infection with these parasites also differed markedly in dogs, ranging from subclinical to peracute. In 1910 a separate species, *Babesia gibsoni* (Patton, 1910), was described from dogs in India [2]. Since the intraerythrocytic trophozoites and meronts of *B. gibsoni* were generally smaller than those of *B. canis* (*sensu lato*), it became common practice to refer to either a large or a small *Babesia* infecting dogs. Molecular characterisation has indicated that *B. gibsoni* falls in the same clade as *B. canis* (*s.l.*) (Clade VI: *Babesia* (*s.s.*)) [3]. The two small *Babesia* spp. that can also cause disease in dogs, i.e. *B. vulpes* Baneth, Cardoso, Brilhante-Simoes & Schnittger, 2019 [4, 5] and *B. conradae* Kjemtrup, Wainwright, Miller, Penzhorn & Carreno, 2006 [6], fall in Clade I (*B. microti*-like) and Clade II (*Babesia* (*s.l.*)), respectively [3]. This review focuses only on *B. canis* (*s.l*.)

For most of the 20th century the proverbial slumber of canines was not disturbed. The causative agent of the disease, wherever it occurred, was commonly referred to as *B. canis*, even though it had been amply demonstrated that different tick vectors were involved, that some parasite stocks were decidedly virulent, while others caused mild or no clinical signs even in splenectomised dogs, and that immunity to one stock did not necessarily protect against another. This was the situation until some 30 years ago, when Uilenberg et al. [7] presented serological evidence and reminded the scientific community that not one but three vector-specific taxa were involved, which they named *B. canis canis*, *B. canis vogeli* and *B. canis rossi*. By 1998 it had been demonstrated that variation in the *18S* rRNA gene segregated genotypes of *B. canis* (*s.l.*) into distinct groups corresponding to the proposed subspecies [8]. Opinion varied as to whether these taxa should be regarded as subspecies of *B. canis* or distinct species. There is no universally accepted criterion (% sequence difference) for classifying organisms to species level based on variation in the *18S* rRNA gene. A recent proposal was that the genetic distance between proposed novel species and their closest described relatives should be greater than the genetic distance between the next two most closely related species [9]. For the purposes of this review, the three taxa are regarded as species. The three taxa are: (i) *Babesia canis* (*s.s.*), transmitted by *Dermacentor reticulatus* and historically confined to Europe, but recently reported from China [10]; (ii) *Babesia rossi* (Nuttall, 1910), transmitted by *Haemaphysalis elliptica* (and possibly *H. leachi*) and confined to sub-Saharan Africa; and (iii) the cosmopolitan *Babesia vogeli* (Reichenow, 1935), transmitted by *Rhipicephalus sanguineus*.

Why was the early research that clearly indicated major biological differences between various canine *Babesia* stocks overlooked or ignored for 50 years? In this review early records and descriptions of the disease in Africa, Europe and Asia are traced and identification of the specific vectors involved is documented. Evidence is also given of the growing realisation that there were substantial biological differences between various stocks (following Uilenberg et al. [7], this term is used rather that strain or isolate). Etymological provenance for *B. vogeli* is proposed.

## South Africa

The parasite concerned is regarded as *B. rossi*, although *B. vogeli* could also be involved [11]. Concurrent infection with *B. rossi* and *B. vogeli* has been reported [12].

### Early records

Shortly after arriving in Cape Town in May 1797, Lady Anne Barnard recorded in her journal that dogs brought from England to South Africa and allowed to roam freely generally did not live long in their new home [13]. In contrast, English dogs that were tied up usually survived. Such precautions were not entirely successful, as in November 1797 she referred to a Pointer as their “only English dog that survived the ailment which attacks all who arrive here” [14]. Mortalities occurring in newly imported dogs which had roamed in the indigenous vegetation around Cape Town suggest a vector-borne infection. Being the most common canine tick-borne disease in the area, babesiosis is a prime candidate.

There is anecdotal evidence of canine babesiosis cases in the Eastern Cape Province in the 1840s, Cape Town area in the 1870s and KwaZulu-Natal in the 1880s [14]. Hutcheon, a colonial veterinarian, first encountered canine babesiosis when posted to Port Elizabeth, Cape Colony, in 1885 [15]. In 1893 he described in some detail the clinical signs, including haemoglobinuria, of what he called “malignant jaundice or bilious fever” [16]. The disease was quite common in coastal towns and districts of the Cape Colony, but rarer in higher-lying inland regions. He noted that the disease bore a close resemblance to red-water or Texas Fever of cattle. By 1899 Hutcheon [15] referred to the local disease as “malignant malarial fever of the dog” and stated that it was widespread in the Cape Colony and adjoining states.

Working in Cape Town in 1899, Spreull [17] successfully subinoculated the infection by means of intravascular and subcutaneous injection of blood from a sick dog to healthy ones. Blood smears were sent to Dr Carrington Purvis in Grahamstown, Eastern Cape Province, who confirmed the presence of pyriform intra-erythrocytic organisms bearing a strong resemblance to the “now well-known redwater organism” [17]. Purvis identified the same piroplasm on blood smears from a dog at Grahamstown, showing similar clinical signs [17]. Following Spreull, Robertson [18] performed numerous inoculation experiments, by both subcutaneous and intravenous injection of infected blood.

In 1901 Robertson [18] stated that the causative organism of the disease in South Africa is without doubt a double pyrosoma, resembling “*Pyrosoma bigeminum*”. By 1904, Lounsbury [14] was referring to the causative organism as *Piroplasma canis*.

### Pathogenicity

Hutcheon [15] regarded babesiosis the most fatal disease affecting dogs in the Cape Colony. Obtaining blood from a sick dog, Robertson [18] successively subinoculated 13 dogs, all of which succumbed to the disease. Citing Robertson (1902), Lounsbury [14] stated that “malignant jaundice” was the most serious and one of the most common diseases affecting dogs in the Cape Colony. Some dogs die within 24 hours of the onset of clinical signs, while most succumb after a few days. The few dogs that survive take weeks or months to recover fully. In a more recent experiment, five dogs artificially infected with *B. rossi* developed parasitaemias usually much higher than 1% and required chemotherapeutic treatment [19]. In contrast to dogs artificially infected with *B. canis* (*s.s.*), clinical signs in these dogs were correlated with peripheral parasitaemia and not with parameters of the coagulation system [19].

Canine babesiosis remains of great clinical importance in South Africa. During the 6-year period 1988–1993, a mean of *c*.10,710 sick dogs were presented *per annum* at the Outpatients Clinic of the Onderstepoort Veterinary Academic Hospital (OPVAH), Pretoria, South Africa [20]. Canine babesiosis was diagnosed in 11.7% of these dogs, 31.4% of the latter requiring hospitalisation for intensive supportive treatment. Of 320 *B. rossi*-infected dogs admitted to OPVAH during 2006–2016, 34 (10.6%) died as a result of the disease within 24 hours of admission. Survivors required intensive treatment, e.g. diminazene aceturate, blood transfusion, crystalloid fluids and a prokinetic drug [21].

### Immunity

When initially reporting on “malignant jaundice or bilious fever” in the Cape Colony in 1893, Hutcheon [16] stated that imported, well-bred dogs were more susceptible to infection than local dogs, which suggests a degree of immunity in the local dogs. By 1902 it had been conclusively proven experimentally that dogs that recovered from the disease did not develop clinical signs when subsequently challenged through tick transmission or subinoculation with infected blood [22]. Immunity was not permanent, however. Blood of recovered dogs harboured piroplasms in their blood for extended periods, up to two years [22]. Dogs that were subclinically infected carriers usually did not develop clinical signs when reinoculated with the parasite [23]. In 1904 Theiler conducted a series of experiments on immunity [24]. He concluded that dogs do not have an innate immunity against babesiosis; serum of surviving dogs that were repeatedly re-infected was protective when injected into susceptible dogs [24].

There was no reduction in virulence after serial passage in dogs by subinoculation, even after 47 passages [23]. Attempts at developing a vaccine by attenuation through subinoculation were therefore futile [23]. A breakthrough came in 1909 when it was discovered that trypan blue, injected intravenously, effected clinical cure while not eliminating the parasite entirely [25]. “Infection and treatment” was therefore proposed as a way to render dogs immune against the disease [23]. Although it is currently generally not the first drug of choice for canine babesiosis, after 110 years trypan blue must be one of the oldest drugs that is still available commercially in veterinary practice.

In 1913 Laveran & Nattan-Larrier [26] challenged two dogs that were immune against the South African stock with a French stock; they developed mild clinical signs, but both survived.

### Identifying the vector

In a series of experiments, published in 1901, Robertson [18] and Lounsbury [27] demonstrated that *Haemaphysalis leachi* (*s.l.*) could transmit the infection. They determined that adult female ticks engorging on sick dogs became infected, passing the infection transovarially to the ensuing larvae. The infection was not transmitted by the larvae and nymphs, but only by adult ticks of the next generation, a phenomenon that had not been reported before. Lounsbury [14] followed this up by further transmission experiments, in which he demonstrated that a healthy dog that had survived the disease remained a subclinical carrier and could infect ticks. In 1902 Lounsbury [14] sent infected nymphs to Nuttall at Cambridge University, UK, where extensive investigations were conducted [23, 25, 28]. Since *H. leachi* (*s.s.*) does not occur in South Africa [29], *Haemaphysalis elliptica* must have been the tick involved. In 1938 Brumpt [30] demonstrated that *R. sanguineus* did not transmit the South African stock. This was confirmed by Lewis et al. [31].

## Elsewhere in sub-Saharan Africa

In the course of a wide-ranging research visit to India and Africa in the late 1890s, Koch cited by Nuttall [28] reported seeing organisms similar to those causing human malaria in the blood of a dog in Dar es Salaam, Tanzania. In 1900, Marchoux [32] found *Piroplasma canis* in blood smears of 11 dogs in Senegal showing no clinical signs other than a slight rise in temperature. This suggests that *B. vogeli* rather than *B. rossi* may have been involved. Marchoux’s report contains detailed illustrations of large pyriform and other piroplasms [32].

## Europe

The parasite concerned is regarded as *B. canis* (*s.s.*), although *B. vogeli* could also be involved.

### Early records

In his 1893 report on “malignant jaundice or bilious fever”, Hutcheon [16] stated that the disease was much more common in the Cape Colony than in Europe, but furnished no details. The first definite records were from Italy, where Piana & Galli-Valerio [1] in 1895 gave a good description of the clinical signs and related pathology of dogs becoming ill. On blood smears from a Pointer dog that became sick after hunting in the hills and on irrigated pastures near Milan they found intra-erythrocytic parasites resembling those described by Smith & Kilbourne [33] from cattle suffering from Texas fever or redwater; this dog survived [34]. Piana & Galli-Valerio named these parasites *Pyrosoma bigeminum* var. *canis* [1]. Soon afterwards, the same disease was reported in dogs that had hunted in marshes near Rome [35].

In a brief report published in 1900 [36], Leblanc, who worked in Lyon, France, stated that he had found many haematozoa resembling those in blood of cattle and sheep with haemoglobinaemia in the blood of a dog suffering from “infectious icterus”. In a subsequent paper [37] he stated that the organisms he saw resembled *P. canis* described by Marchoux from dogs in Senegal [32]. In 1901 Nocard & Almy [38] reported haemoglobinuria in a dog and found piroplasms resembling those causing Texas fever in a blood smear. They successfully passed the infection to another dog by intravenous injection. Soon afterwards, Almy [39] reported 5 further cases presented at the veterinary clinic at Alfort, France.

### Pathogenicity

In their pioneering investigation in 1902, Nocard & Motas [40, 41] performed numerous experiments in France, stating that 63 of their infected dogs had died. They distinguished between two distinct manifestations: acute and chronic. In the acute form, death usually ensued 2 or 3 days after clinical signs appeared. Onset was much slower in the chronic form, and the dog may be sick for up to 30–60 days; most of these patients recovered. A significant finding was that subinoculation of blood from dogs suffering acute disease led to acute disease in the recipients; similarly, subinoculation of blood from dogs suffering from the chronic forms of the disease led to a chronic manifestation in the recipients. Since Nocard & Motas [40, 41] did not specify the origin of the piroplasms they were working with, this observation may indicate that they used more than one stock in their experiments. A further interesting statement is that, in the acute manifestation, the organisms appeared to be smaller (see “Discussion”). Nocard & Motas [40, 41] also pointed out that artificially infected pups (2–12 weeks of age) reacted more severely than older dogs and invariably succumbed to the disease.

In a more recent experiment, 5 dogs artificially infected with *B. canis* (*s.s.*) showed transient parasitaemia, usually < 1%, low packed cell volume values and congestion of internal organs [19]. Clinical signs were not correlated with parasitaemia, but with effects on the coagulation system [19].

### Immunity

Nocard & Motas [40, 41] reported that protective immunity developed in dogs that recovered from the disease. Dogs injected with a mixture of virulent blood and blood from recovered dogs remained healthy. Recovered dogs challenged with infective doses much larger than doses invariably fatal in control dogs remained refractory. In contrast to the situation pertaining in South Africa, Nocard & Motas [40, 41] claimed that immunity was permanent, but did not present supporting evidence.

In 1913, Laveran & Nattan-Larrier [26] challenged 7 dogs that were immune against the French stock with the South African stock; 6 of the dogs died. In 1938, Brumpt [30] reported that a dog that survived infection with both the Moroccan and European stock succumbed to experimental infection with the South African stock.

### Identifying the vector

*Ixodes ricinus* (reported as *I. reduvius*) was the only tick infesting the dog from which *B. canis* (*s.s.*) was described [1]. Suspecting that it might be a vector, Piana & Galli-Valerio [34] examined gut content of the ticks, but did not find piroplasms. *Dermacentor reticulatus* was the only tick infesting sick dogs at Alford, France [40, 41]. In an experiment, larvae hatched from eggs laid by ticks engorging on sick dogs failed to transmit the infection [40, 41]. It was only in 1919 that Brumpt [42] demonstrated that *D. reticulatus* was a vector in Europe, and that the infection was transmitted transovarially by adult female ticks engorging on infected dogs. Adult ticks of the ensuing generation passed the infection to the dog that they fed on; the preceding larvae and nymphs were apparently not infective. In a later study, Regendanz & Reichenow [43] confirmed that transovarially transmitted infection was not passed to dogs by larvae of the next generation; in contrast to Brumpt’s findings [42], nymphs were capable of transmitting the infection.

In 1931, Nieschulz & Wawo-Roentoe [44] reported unsuccessful attempts at transmitting European *B. canis* to splenectomised dogs by *R. sanguineus*. Since the *Babesia* laboratory stock used had undergone at least 100 tick-free passages, the authors cautioned that it may have lost its infectivity to ticks. Unfortunately, they did not use *D. reticulatus* as a control [44]. In 1932, Regendanz & Reichenow [43] and in 1938 Brumpt [30] confirmed that *R. sanguineus* did not transmit European *B. canis*. Brumpt [30] further demonstrated that, under experimental conditions, *H. elliptica* transmitted European *B. canis* transstadially: nymphs engorging on infected dogs transmitted the infection in the adult stage (see “Discussion”).

## North Africa and Asia

The parasite involved is regarded as *B. vogeli*.

### Early records

In 1901, Almy [39] reported on a Fox Terrier that had fallen ill in Tunisia a few weeks previously. When presented at the veterinary clinic in Alfort, France, the dog was severely anaemic and depressed. It died two days later. Autopsy revealed splenomegaly and “piroplasma bigeminum” in the blood. In 1903, Martini, cited by Nuttall [28], gave an unsubstantiated report from Egypt.

In India, “canine malaria” was first reported in 1904 by Dalgetty [45], who described intra- and extraerythrocytic organisms in a Fox Terrier with clinical signs including haemoglobinuria. In 1905, James [46] mentioned seeing *P. canis* on a blood smear of a dog. In 1906, Webb [47] attributed high mortality in well-bred fox-hounds in India to piroplasmosis, but some other disease was probably the primary cause. Although blood smears were “frequently examined” (no details given), piroplasms were not detected in specimens from any of the sick dogs, but only at *post-mortem* examination of a single dog. The accompanying illustration confirmed the presence of round to oval and pear-shaped piroplasms [47]. In 1907, Christophers [48] stated that canine piroplasmosis was endemic among native dogs of India. In his paper naming and describing *B. gibsoni*, Patton [2] mentioned that in 1907 the entire pack of Foxhounds of the Madras Hunt in India had died of *P. canis* infection. This seems unlikely, and no details were given to substantiate the statement.

Reporting in 1908 on the occurrence of ovine piroplasmosis in Qingdao, China (at that time a German-controlled port), Eggebrecht [49] mentioned that they were chronic-type infections, as he had seen in a number of dogs artificially infected with blood containing a dog “pirosome”. Eggebrecht did not state explicitly that this had been in China. Reporting on parasitic infections in humans and animals in Tonkin (Hanoi area, Vietnam), Mathis & Léger [50] reported in 1911 that *P. canis* was frequently encountered, both in local and imported dogs. The stock was taken to France, where further experiments were conducted [51, 52]. The presence of *B. vogeli* in northern Vietnam has been confirmed [53].

### Pathogenicity

Working in France, Ciuca [51] demonstrated that the *Babesia* stock brought from Tonkin was mildly pathogenic, except for young pups: all 2–4-week-old pups succumbed after experimental infection. Life-threatening clinical signs in naturally infected young pups have also been reported from Italy [54] and the USA [55, 56].

Older dogs experimentally infected by Ciuca [51] generally showed mild clinical signs but survived. This included 10 of 14 splenectomised dogs. Most of 8 adult dogs naturally infected with *B. vogeli* showing clinical signs of babesiosis had concomitant diseases or predisposing factors, e.g. leishmanosis, splenectomy, chronic renal insufficiency or immunosuppressive doses of corticosteroids [54]. When babesiosis was diagnosed in five 11–18-day-old Greyhound pups at a kennel in the USA, none of the 107 adult dogs at the same kennel had a history of babesiosis, but 63 (58.9%) had positive IFAT titres [55], suggesting sub-clinical infections.

In a recent study, 6 healthy Beagles (3 having been splenectomised) experimentally infected with a Chinese stock of *B. vogeli* showed fever, partial anorexia and malaise [57]. Clinical signs were more severe in the splenectomized dogs, two of which required treatment.

### Immunity

In 1913, Ciuca [52] reported that a dog that survived infection with the Tonkin stock remained a subclinical carrier of the piroplasm for at least 9 months. When challenged with the South African stock 28 months later, the dog succumbed to the disease. Brumpt [30] reported that a dog surviving infection with both a Moroccan and a European stock succumbed when challenged with the South African stock.

### Identifying the vector

In the outbreak among Foxhounds in India, mentioned above, the dogs’ bedding straw was removed and they slept on bare wooden benches [47]. Although this did not prevent infection entirely, the rate of progression of infection was reduced. This suggests that kennel ticks (*R. sanguineus* (*s.l.*)) were the vectors involved. In 1907, Christophers [48], also working in India, published a preliminary note on the development of *P. canis* in *R. sanguineus*. He confirmed transovarial transmission in infected female ticks; although next generation larvae were apparently unable to pass the infection, the nymphs and adults certainly could. Christophers [48] also stated that nymphs infected while engorging could transmit the infection as adults.

According to Wenyon [58], James [46] who had reported *P. canis* in a dog in Assam, India, in 1905 took *R. sanguineus* to England and successfully infected dogs. Wenyon [58] also took *R. sanguineus* from Aleppo, Syria, to England where the ticks infected a dog 6 months later.

In 1910, Brumpt [59], working in France, received an infected dog from Tunisia. About 50 female *R. sanguineus* ticks engorging on this dog gave rise to thousands of larvae, which were used in various transmission experiments. Brumpt [59] confirmed transovarial transmission of *Babesia*, but found that only adult ticks and not larvae and nymphs transmitted the infection. Brumpt [59] could also not confirm Christophers’s [48] claim that *R. sanguineus* nymphs engorging on infected dogs transmitted the infection as adults.

## Growing realisation of substantial differences between various stocks

As early as 1904, Nuttall [28] cautioned that there should be a clear distinction between observations made in Europe and in Africa, since parasites in different localities may not necessarily be the same. In the same year Lounsbury [14] commented that there must be more than one vector involved since *H. elliptica*, a vector in South Africa, did not occur in Europe.

Based on their cross-immunity experiments, Laveran & Nattan-Larrier [26] commented in 1913 that they were led to conclude that the South African stock constitutes, if not a species, at least a variety quite distinct from the French one.

After they could transmit European *B. canis* (*s.s.*) by *D. reticulatus* but not by *R. sanguineus*, Regendanz & Reichenow [43] stated in 1932 that they suspected that there are variants of *B. canis* that only differ in that they are adapted to different tick vectors. In 1935, Reichenow [60] proposed a new species name for the stock transmitted by *R. sanguineus*. In 1938, Brumpt [30] concluded that, although morphologically indistinguishable, different *B. canis* stocks had different antigenic properties; cross-immunity did not occur.

By the 1930s it was widely known that there were vector-specific stocks, and that stocks from various geographical regions differed markedly in their virulence. This information had been published in some of the leading scientific journals of the day. The reason why this well-documented knowledge was subsequently overlooked for 50 years remains a mystery.

## Taxonomy

### Genus names

When Victor Babes discovered piroplasms in blood of anaemic cattle in Romania in 1888, he was under the impression that they were bacteria and named them *Haematococcus bovis* [61]. Starcovici, a student of Babes, realised that they were protozoans and in 1893 created the genus *Babesia*, in honour of the discoverer [62]. More or less at the same time, Smith & Kilbourne [33] named the causative agent of Texas fever *Pyrosoma bigeminum*. Realising that the genus name *Pyrosoma* (or *Pirosoma*) was preoccupied, Patton in 1895 named the organism *Piroplasma bigeminum* [63]. Citing its morphological similarity to the causative agent of Texas fever, Piana & Galli-Valerio [1] named the organism they identified from a dog in Italy in 1895 *Pyrosoma bigeminum* var. *canis*. By 1900 this organism was referred to as *Piroplasma canis* [32]. This name was in general use until 1918, when du Toit, pointing out that *Babesia* was the senior name, relegated *Piroplasma* to subgenus status and listed the parasite as *Babesia canis* [63].

### Species names and etymological provenance

#### *Babesia canis*

When describing and naming “*Pyrosoma bigeminum* var. *canis*” in 1895, Piana & Galli-Valerio [1] followed convention by naming the parasite after the host in which it was first encountered.

#### *Babesia rossi*

**Fig. 1:**
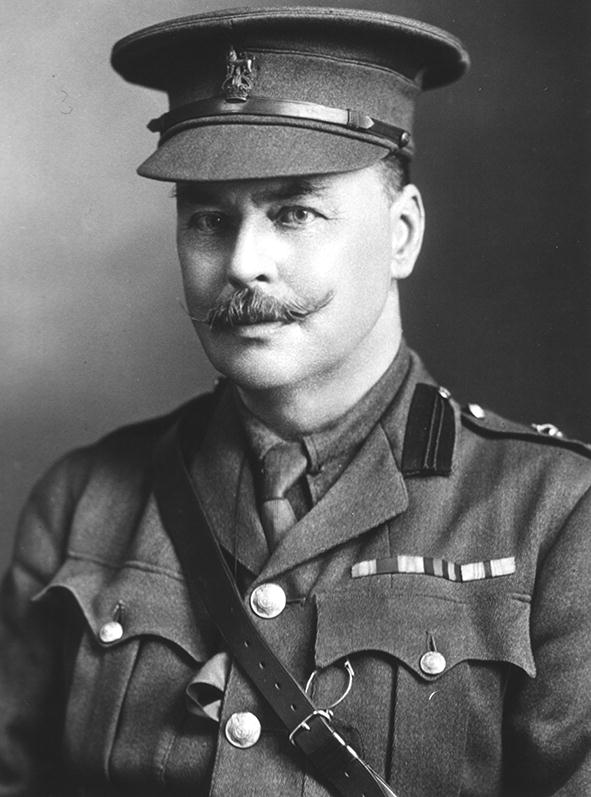
Sir Ronald Ross (1857–1932). Recipient of the Nobel Prize for Physiology or Medicine, 1902. (courtesy: US National Library of Medicine)

*Babesia canis rossi* was the name assigned in 1989 by Uilenberg et al. [7] to the virulent piroplasm infecting dogs in sub-Saharan Africa. In a footnote in which he described *Piroplasma rossi* from a side-striped jackal (*Canis adustus*) in Kenya in 1910, Nuttall [64] stated that the name was in honour of Professor Ronald Ross, C.B., F.R.S. In a subsequent paper, Nuttall [65] created the genus *Rossiella* for this species, but it did not gain general acceptance [63]. Sir Ronald Ross (1857–1932) (Fig. 1) was a British Army doctor who played a major role in proving that mosquitoes transmitted human malaria; he was awarded the Nobel Prize for Physiology or Medicine in 1902 [66, 67]. Uilenberg et al. [7] were justified in using this name, since it has subsequently been shown that black-backed jackals (*Canis mesomelas*) are natural reservoir hosts of *B. rossi* and that its only proven vector, *H. elliptica*, is the most prevalent tick infesting black-backed jackal populations [68, 69].

#### *Babesia vogeli*

**Fig. 2:**
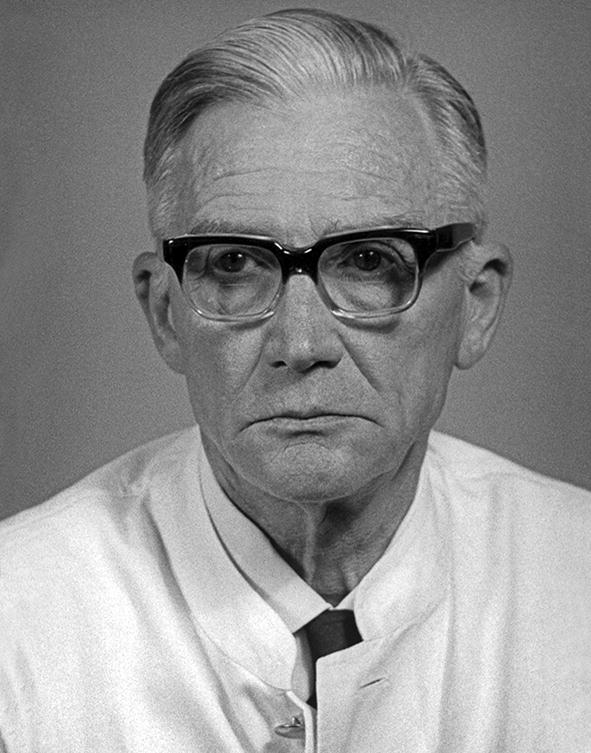
Dr Hans Vogel (1900–1980). Helminthologist; director of the Bernard Nocht Institute (1963–1968). (courtesy: Bernard Nocht Institute, Hamburg Germany)

In his research on the stock transmitted by *R. sanguineus*, Reichenow [60] described the parasite as being larger than that transmitted by *D. reticulatus*, and named it *Babesia major*. He based his description on an East Asian stock, but did not mention its origin. In a subsequent paper on *Theileria parva*, Reichenow [70] acknowledged in an aside that *B. major* had previously been assigned to a bovine piroplasm [71] and merely stated that he therefore named the organism *B. vogeli*. At that time Reichenow was working at the Institute for Maritime and Tropical Diseases (renamed Bernard Nocht Institute for Tropical Medicine in 1942) in Hamburg, Germany [72]. Hans Vogel, a helminthologist working at the same institute, discovered the developmental cycle of *Opisthorchis felineus*, a zoonotic fluke, and later proved that hosts can acquire immunity to schistosomiasis [72]. Vogel visited China in the early 1930s. The *B. gibsoni* stock at the Institute in Hamburg originated from the Shanghai region of China [30]. It was inoculated to a dog, which was entrusted to Dr Vogel who was returning to Hamburg. During the sea voyage, two dogs were consecutively subinoculated, and since 1934 the stock was maintained at the Institute for Maritime and Tropical Diseases by dog-to-dog passage. Although Reichenow [70] did not state the reason why he named the parasite *B. vogeli*, there can be little doubt that it was named for Hans Vogel. Later, the Neotropical taeniid *Echinococcus vogeli* was also named in his honour [73]. Hans Vogel (1900–1980) (Fig. 2) worked primarily on zoonotic flukes and tapeworms. He concluded his career as director of the Bernard Nocht Institute, 1963–1968.

## Discussion

The last of the early papers on the identity and transmission of these canine babesias was published in 1938 [30]. In retrospect, it seems incomprehensible that this relatively large body of research, generally published in leading journals, was ignored for the next 50 years. A contributing factor may be that the titles of the two papers in which *B. vogeli* was named gave no indication thereof [60, 70]. The title of the first paper [60] merely refers to transmission and development of piroplasms; the title of the second paper refers to the development of *Theileria parva* in *Rhipicephalus appendiculatus* [70].

In subsequent decades, *B. canis* (*s.l.*) was used indiscriminately when referring to large canine piroplasms. Caution should therefore be exercised when this literature is assessed. Most of the research during this period focused on treatment and aspects of disease manifestation.

Did Nocard & Motas [40, 41] use stocks of both *B. canis* (*s.s.*) and *B. vogeli* in their experiments? They distinguished between an acute and a chronic manifestation of the disease and stated that subinoculation of blood from such cases, respectively, consistently gave the same results. Nocard & Motas [40, 41] mentioned that erythrocytes generally contained a single, large circular parasite; erythrocytes with multiple parasites, which were smaller, were especially found in the acute manifestation. Could this indicate *B. canis* (*s.s.*)? *Babesia vogeli*, the least virulent species, was initially named *B. major*, since Reichenow [60] regarded the parasites as being somewhat larger than *B. canis* (*s.s.*), but no actual measurements were given. On the other hand, two subgroups (A and B) were distinguished by restriction fragment length polymorphism analysis and direct sequencing of a 559-bp region of the *18S* rRNA gene of *B. canis* (*s.s.*) [74]; subgroup B was regarded as the more pathogenic of the two [75].

The finding by Brumpt [30] that *H. elliptica* nymphs engorging on *B. canis*-infected dogs under experimental conditions could transmit the infection as adults is probably of very little, if any, practical importance. Under experimental conditions all stages of *H. elliptica* readily engorge on dogs [31]. Under natural conditions, *H. elliptica* larvae and nymphs feed on small mammals; adults prefer large carnivores [76]. It is highly unlikely, therefore, *B. canis* (*s.s.*) would become established naturally by transstadial transmission if subclinical carrier dogs were brought into *H. elliptica*-endemic areas.

It is interesting to note that researchers working under less than ideal conditions in outposts of the British Empire had identified the vector of *B. rossi* by 1901 and that of *B. vogeli* by 1907. More than a decade elapsed before the vector of *B. canis* (*s.s.*) was confirmed in Europe, where state-of-the-art research facilities were available.

## Conclusions

Pioneering research published between 1895 and 1938 clearly demonstrated that *B. canis* (*s.l.*) was actually three distinct, vector-specific entities. For some unknown reason, this substantial body of knowledge was overlooked or ignored for 50 years. Caution should be exercised when assessing literature on clinical manifestation, treatment and prevention of canine babesiosis/piroplasmosis published between 1940 and 1989 (and beyond), since the causative organism is usually merely referred to as *B. canis*. Since the cosmopolitan *B. vogeli* is the least pathogenic of the three entities, clinical reports from Europe can safely be attributed to *B. canis* (*s.s.*), while such reports from sub-Saharan Africa can be attributed to *B. rossi*. Elsewhere in the world, it can be assumed that *B. vogeli* is probably involved. Lastly, may this review reinforce recognition of the importance of being thoroughly conversant with the relevant literature on specific topics. Check the original sources. Don’t let sleeping dogs lie.

## Data Availability

Not applicable.
